# Joint association of sedentary time and physical activity with abnormal heart rate recovery in young and middle-aged adults

**DOI:** 10.1186/s12889-024-19298-9

**Published:** 2024-07-04

**Authors:** Zhizhen Liu, Peiyun Zheng, Yong Fang, Jie Huang, Jia Huang, Liming Chen, Qiaoling Hu, Chunyan Zou, Jing Tao, Lidian Chen

**Affiliations:** 1 National-Local Joint Engineering Research Center of Rehabilitation Medicine Technology, Fujian University of Traditional Chinese Medicine, Fuzhou, 350122 China; 2 College of Rehabilitation Medicine, Fujian University of Traditional Chinese Medicine, Fuzhou, 350122 China; 3 Health Management Center, The Second People’s Hospital Affiliated to Fujian University of Chinese Medicine, Fuzhou, 350003 China

**Keywords:** Sedentary time, Physical activity, Heart rate recovery, Cardiac autonomic function

## Abstract

**Background:**

Abnormal heart rate recovery (HRR), representing cardiac autonomic dysfunction, is an important predictor of cardiovascular disease. Prolonged sedentary time (ST) is associated with a slower HRR. However, it is not clear how much moderate-to-vigorous physical activity (MVPA) is required to mitigate the adverse effects of sedentary behavior on HRR in young and middle-aged adults. This study aimed to examine the joint association of ST and MVPA with abnormal HRR in this population.

**Methods:**

A cross-sectional analysis was conducted on 1253 participants (aged 20–50 years, 67.8% male) from an observational study assessing cardiopulmonary fitness in Fujian Province, China. HRR measured via cardiopulmonary exercise tests on a treadmill was calculated as the difference between heart rate at peak exercise and 2 min after exercise. When the HRR was ≤ 42 beats·minute^−1^ within this time, it was considered abnormal. ST and MVPA were assessed by the IPAQ-LF. Individuals were classified as having a low sedentary time (LST [< 6 h·day^−1^]) or high sedentary time (HST [≥ 6 h·day^−1^]) and according to their MVPA level (low MVPA [0–149 min·week^−1^], medium MVPA [150–299 min·week^−1^], high MVPA [≥ 300 min·week^−1^]). Finally, six ST-MVPA groups were derived. Associations between ST-MVPA groups with abnormal HRR incidence were examined using logistic regression models.

**Results:**

53.1% of the young and middle-aged adults had less than 300 min of MVPA per week. In model 2, adjusted for possible confounders (e.g. age, sex, current smoking status, current alcohol consumption, sleep status, body mass index), HST was associated with higher odds of an abnormal HRR compared to LST (odds ratio (OR) = 1.473, 95% confidence interval (CI) = 1.172–1.852). Compared with the reference group (HST and low MVPA), the HST and high MVPA groups have a lower chance of abnormal HRR (OR, 95% CI = 0.553, 0.385–0.795). Compared with individuals with HST and low MVPA, regardless of whether MVPA is low, medium, or high, the odds of abnormal HRR in individuals with LST is significantly reduced (OR, 95% CI = 0.515, 0.308–0.857 for LST and low MVPA; OR, 95% CI = 0.558, 0.345–0.902 for LST and medium MVPA; OR, 95% CI = 0.476, 0.326–0.668 for LST and high MVPA).

**Conclusion:**

Higher amounts of MVPA appears to mitigate the increased odds of an abnormal HRR associated with HST for healthy young and middle-aged adults.

**Supplementary Information:**

The online version contains supplementary material available at 10.1186/s12889-024-19298-9.

## Introduction

Heart rate recovery (HRR) refers to the heart rate decrease observed after exercise, which reflects cardiac autonomic function [1–3]. This physiological phenomenon can be mainly attributed to parasympathetic reactivation [3]. Interestingly, an abnormal HRR (a reduction of less than 42 beats after 2 min of peak exercise cessation) can indicate an impaired parasympathetic reactivation, which is, in turn, an independent predictor of cardiovascular disease (CVD) and mortality [4, 5].

Previous research has demonstrated that certain unhealthy lifestyle-related factors, such as short-term sleep deprivation [6], smoking [7], being overweight [8], and physical inactivity [9], are linked with a slower HRR. Concerning physical inactivity, not only the influence of physical exercise on health outcomes has been extensively discussed in the last years, but also the influence of daily sedentary time (ST). Sedentary behaviour harms cardiovascular health and may be associated with impaired cardiac autonomic function [10, 11]. Higher levels of ST have been shown to be associated with poorer cardiac autonomic function as measured by heart rate variability in young and middle-aged adults [12, 13]. Therefore, investigating the relationship between sedentary behavior and an abnormal HRR can have significant clinical implications for the early prevention of CVD in healthy young and middle-aged adults.

Epidemiological studies have indicated that sedentary behavior is becoming increasingly common among young and middle-aged adults [14, 15]. Previous research on the association between sedentary behavior and HRR has primarily focused on adolescents [16] and patients with chronic obstructive pulmonary disease (COPD) [17]. In healthy men, the HRR was slower in the sedentary group that self-reported no leisure time physical activity (PA) than in the walking and running groups [9]. Studies that investigated healthy adults generally defined sedentary behavior as the lowest level of PA [8, 9], that is, no leisure PA time. Thus, less attention was paid to the effect of daily ST on HRR in healthy young and middle-aged adults.

To reduce the health risks associated with prolonged sedentary activity, the WHO guidelines recommend that adults accumulate the recommended levels of PA [18]. High levels of PA have been found to attenuate or even offset the risk of CVD and mortality in sedentary adults [19, 20]. In this sense, young and middle-aged adults with adequate levels of moderate-to-vigorous physical activity (MVPA) have an increased HRR [21]. On the other hand, sedentary behavior has been associated with slower HRR in healthy adult [9, 22]. However, it remains unclear how much MVPA can attenuate or even mitigate the adverse impact of a sedentary lifestyle on the HRR in healthy young and middle-aged adults.

We hypothesizes that individuals with a longer ST have higher odds of an abnormal HRR, and MVPA may be a protective factor for HRR. Therefore, the aim of this study was to assess the joint association of daily ST and MVPA with an abnormal HRR in healthy young and middle-aged adults aged 20–50 years.

## Methods

### Study design and participants

This cross-sectional study was conducted in Fujian Province, China, as part of the Observational Study to Assess the Functional Status of Chinese People project, which aims to explore the factors influencing cardiorespiratory fitness in young and middle-aged adults. From 2013 to 2020, individuals who came to the medical examination center of the Second People's Hospital of Fujian University of Traditional Chinese Medicine for health checks were informed of the study information and were asked if they wished to participate in the study. Participants were considered eligible if they: 1) were 20–50 years old, 2) had no diagnosis of chronic diseases such as CVD (heart disease, hypertension, stroke, peripheral vascular disease, arteriosclerosis), pulmonary diseases (asthma, pulmonary emphysema, bronchopneumonia, active stage of tuberculosis), diabetes, hyperthyroidism, hypothyroidism, osteoporosis, and cancer. 3) did not present abnormalities on electrocardiogram (ECG) examination, 4) self-reported not taking anti-arrhythmic drugs, antihypertensive drugs, hypoglycemic drugs, lipid-lowering drugs and psychotherapeutic drugs.

Selected participants were asked to complete biochemical blood tests and blood pressure measurements on the first day at the Laboratory Department of our Physical Examination Center; on the second day, a cardiopulmonary exercise test and a standard questionnaire assessment were performed by a professional physician at the Exercise Intervention Room of our Physical Examination Center. Participants were asked to fast overnight for 12 h prior to blood collection and 2 h prior to the cardiopulmonary exercise test.

After excluding participants with missing values for PA (*n* = 1310), ST (*n* = 37), HRR and oxygen uptake (VO_2_) (*n* = 89) or any covariates (*n* = 209), a total of 1253 participants were included in the analysis (detailed are shown in the study population flowchart).

The study was approved by the Medical Ethics Committee of the Second Affiliated Hospital of Fujian University of Traditional Chinese Medicine, China (No.SPHFJP-K2019059-02). All participants gave written informed consent.

### Measurements

#### International Physical Activity Questionnaire: ST and MVPA

Sedentary behavior and PA were assessed using the Chinese version of the International Physical Activity Questionnaire-Long Form (IPAQ-LF), which is one of the most widely used questionnaires for measuring PA levels in adults [23], and its validity (correlation of total PA with accelerometer data was 0.35) and reliability (coefficients for vigorous, moderate, walking, and total PA ranged from 0.74 to 0.97) were tested in Chinese population studies [24].

Participants were asked to think about the time they had spent sitting or lying down at work, at home, and during leisure time in the last 7 days. They were asked to write down the number of hours and minutes per day they spent sitting for a weekday and a weekend day. They also were asked to estimate the time spent sitting during travel [25]. Regarding the literature, total weekly sitting time (hours·week^−1^) is defined as the sum of weekday sitting time, weekend sitting time, and transportation sitting time [26]. The average sitting time per day (hours·day^−1^) is calculated by dividing the total sitting time per week by 7. Average sitting time ≥ 6 h·day^−1^ and < 6 h·day^−1^ was defined as high-sedentary time (HST) and low-sedentary time (LST), respectively [27, 28].

Participants were asked sequentially about their PA related to work, transportation trips, housework, and leisure in the past 7 days. Within each category of daily activity, the 1-week frequency (day·week^−1^) and cumulative time per day (minutes·day^−1^) of different intensities of PA were further asked. A metabolic equivalent value (MET) was assigned to each type of activity [25]. The MVPA was assigned a value of ≥ 3.0 MET [29], and the amount of MVPA per week for participants was calculated by summing the cumulative weekly time (1-week frequency multiplied by time per day) for each type of PA (MET ≥ 3.0) in minutes·week^−1^. Based on the World Health Organization's physical activity and sedentary behaviour guidelines [18], the weekly amount of MVPA is classified as follows: low MVPA (0–149 min·week^−1^), medium MVPA (150–299 min·week^−1^), and high MVPA (≥ 300 min·week^−1^) group.

#### Cardiopulmonary exercise test and HRR measurement

Participants were subjected to a cardiopulmonary exercise test (CPET) using the exercise cardiopulmonary function measurement system (JAEGER Master Screen CPX) from Jäger, Germany. The participants were seated for 3 min, wearing a mask, checked for air leaks, and connected to a gas analyzer. Blood pressure, ECG, and oxygen saturation were monitored. After that, they performed incremental exercise on the treadmill according to the modified Bruce protocol. According to this protocol, each stage lasted 3 min. The exercise test started at 2.7 miles per hour (mph) and 0% grade. At stage 3 (within 9 min), the speed of 2.7 miles per hour (mph) was maintained, and the grade was increased by 5% at each stage. From level 4 onward, the intensity was increased by increasing speed (0.6 mph from stage 4 to 5, 1.3 mph from stages 5–7; 0.8 mph from stages 7–9) and grade (2% increment at each stage). The speed and grade were adjusted according to the protocol until the assessment was terminated when there was exhaustion (subjective fatigue level > 17) or discomforts such as chest tightness and chest pain, dyspnea, leg muscle soreness, or manifestations such as decreased ST-T segment on ECG and abnormal blood pressure. The entire test was conducted by specially trained physicians.

Heart rate (HR) and peak oxygen uptake (VO_2peak_, ml·kg^−1^·min^−1^) were measured during the CPET. The peak heart rate (HR_peak_) during exercise and heart rate at 2 min after exercise (HRrec2) were recorded. The difference between these two heart rate values was calculated as the heart rate recovery value (i.e. HRR∆2 = HR_peak_—HRrec2). Heart rate recovery at 2 min (HRR∆2) ≤ 42 beats per minute (beats·minute^−1^) can be used as a reliable criterion for determining abnormal heart rate recovery [4, 5]. VO_2peak_ data was accepted only if the participant met the following criteria: 1) the subject was tired fatigued to continue the exercise, and 2) the respiratory exchange ratio was ≥ 1.1.

#### Covariates

In the morning, after overnight (12-h) fasting, participants were seated and 5 mL of blood was drawn from the elbow vein. A fully automated biochemical analyzer (C16000) from Abbott Ltd. was used to analyze the following parameters: total cholesterol, low-density lipoprotein cholesterol (LDL cholesterol), high-density lipoprotein cholesterol (HDL cholesterol), triglycerides, and fasting glucose. All test kits were purchased from Beijing Lidman Biochemical Co., Ltd. and testing was performed by the Department of Laboratory of the Second People's Hospital of Fujian University of Traditional Chinese Medicine. Body mass index (BMI, kg·m^−2^) was calculated from height and weight. After 10 min of rest, blood pressure was measured three times, and the mean of the two last systolic and diastolic values was retained for analysis. History of heart disease, diabetes mellitus, and other medical and family history was obtained from a disease history questionnaire. In the lifestyle questionnaire, smoking, alcohol consumption and sleep status were assessed and categorized as current smoking: yes/no; current alcohol consumption: yes/no; and late sleep (falling asleep after 23:00): yes/no, respectively.

### Statistical analysis

Statistical analysis was performed using IBM SPSS Statistics (version 26.0) and R software (version 4.3.3). Participant characteristics were described as mean ± standard deviation (SD) or median (interquartile range) for continuous variables and the number of cases (percentage) for categorical variables. Characteristics of the two groups (LST and HST) were compared using using independent two-sample t-tests or two independent samples rank sum tests for continuous variables, and Pearson chi-square tests or non-parametric rank sum tests for categorical variables.

A binary logistic regression model (odds ratio (OR) and 95% confidence interval (95% CI) were was used to assess the relationship between ST,MVPA and the odds of incidence of abnormal HRR. Modeling was conducted as follows: 1) crude model: no covariates were put in; 2) model 1: adjusted for age and sex; 3) model 2: adjusted for age and sex, BMI, fasting glucose, total cholesterol, LDL cholesterol, HDL cholesterol, triglycerides, systolic blood pressure, diastolic blood pressure, smoking, alcohol drinking, sleep status, and family health history (hypertension, diabetes, heart disease). To assess the joint association of ST and MVPA with the odds of developing abnormal HRR, six groups were established from the combination of the ST (LST and HST) and MVPA (low, medium and high) values, with the combined group of HST and low MVPA being used as the reference group. The joint association of ST and MVPA with the odds of abnormal HRR was examined in the same series of models for men and women, separately (See Table S2 and Table S3). The dose–response relationship between MVPA and the odds of abnormal HRR in LST and HST patients was further analysed using a restricted cubic spline regression model with 4 nodes (5th, 35th, 65th, and 95th percentiles). The Wald test is used for nonlinear test. A difference was considered statistically significant at *P* < 0.05.

## Results

**Fig. 1:**
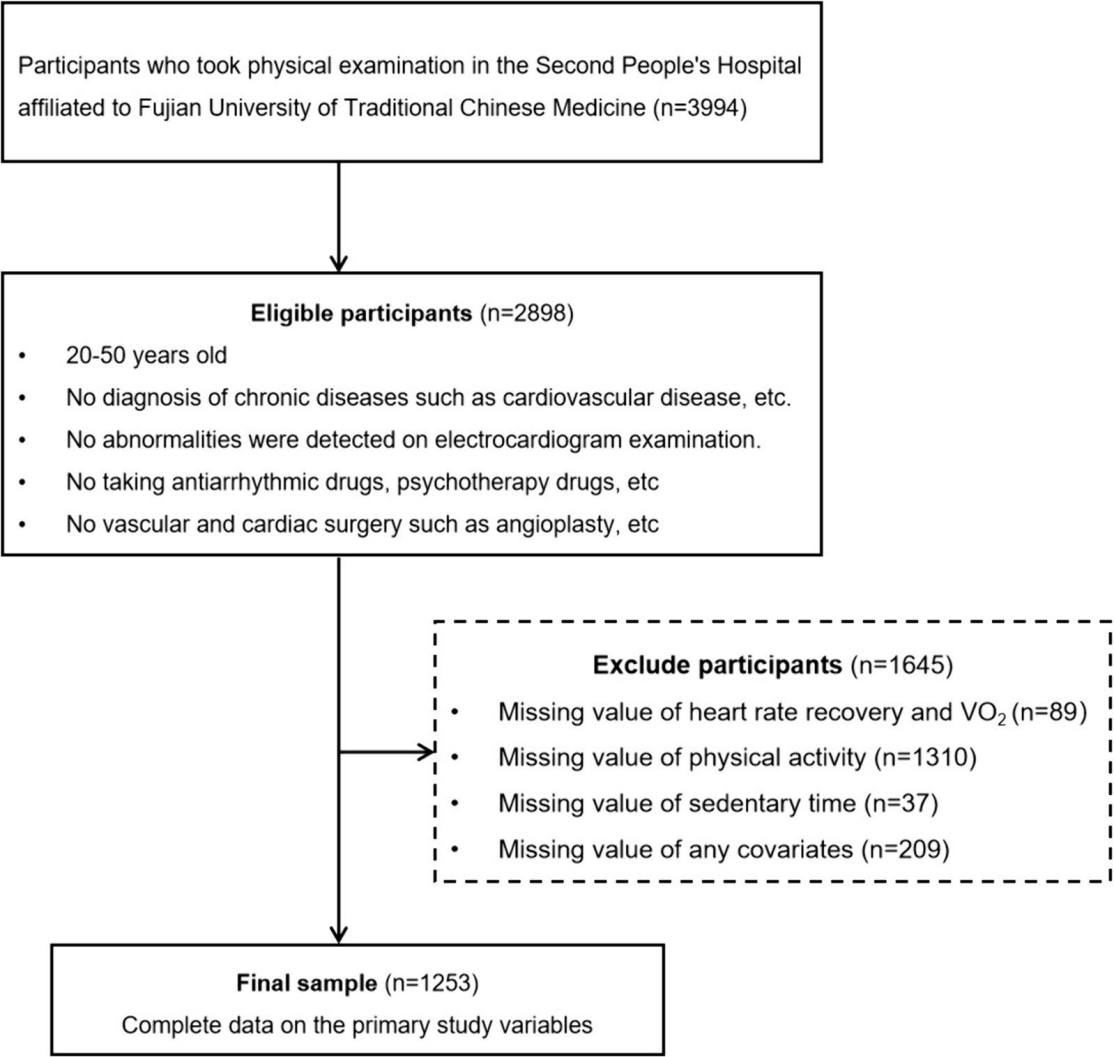
Flow chart of participation

Figure 1 shows the number of participants eligible, excluded, and finally included for analysis in this study. The characteristics of the participants are shown in Table 1. The median value for HRR was 44 beats/min (25th and 75th percentiles, 37 and 50 beats/min). An abnormal HRR of 42 beats/min or less was 49.5% in the HST group. The proportion of late sleep habits in the HST group was higher than that in the LST group. The proportion of low and medium MVPA in the HST group was higher than that in the LST group. The peak heart rate of HST participants was higher than that of the LST group. (Table 1).

**Table 1:** Participant characteristics for the total study population and according to sedentary time category

Variable	Total Population (*n* = 1253)	LST (*n* = 564)	HST (*n* = 689)	*P* value
Demographics				
Age, year	33 (28–40)	33.50 (29–40)	33 (28–39)	0.330
Sex (% male)	849 (67.80)	374 (66.30)	475 (68.90)	0.322
Lifestyle factors				
Current smoking (% yes)	172 (13.70)	77 (13.70)	95 (13.80)	0.945
Current alcohol consumption (% yes)	357 (28.50)	165 (29.30)	192 (27.90)	0.588
Late sleep (% yes)	948 (75.70)	402 (71.30)	546 (79.20)	0.001
Family history (% with positive history)				
Hypertension	531 (42.40)	243 (43.10)	288 (41.80)	0.647
Diabetes	250 (20)	103 (18.30)	147 (21.30)	0.176
Heart disease	77 (6.10)	30 (5.30)	47 (6.80)	0.271
Cardiovascular health factors				
Body mass index, kg·m^−2^	23.27 (20.96–25.56)	23.45 ± 3.20	23.34 ± 3.35	0.555
Plasma glucose, mmol·L^−1^	4.93 (4.69–5.23)	4.95 (4.67–5.23)	4.92 (4.70–5.24)	0.610
Total cholesterol, mmol·L^−1^	4.82 (4.30–5.45)	4.80 (4.30–5.45)	4.85 (4.29–5.45)	0.988
Low-density lipoprotein cholesterol, mmol·L^−1^	2.87 (2.40–3.41)	2.88 (2.42–3.38)	2.87 (2.40–3.42)	0.959
High-density lipoprotein cholesterol, mmol·L^−1^	1.26 (1.08–1.46)	1.25 (1.09–1.46)	1.26 (1.07–1.46)	0.945
Triglycerides, mmol·L^−1^	1.06 (0.77–1.58)	1.07 (0.77–1.62)	1.06 (0.77–1.56)	0.756
Systolic blood pressure, mm Hg	118 (110–128)	119 (111–128)	119 (110.50–128)	0.994
Diastolic blood pressure, mm Hg	71 (65–79)	72 (66–79)	71 (64.50–79)	0.439
Cardiorespiratory fitness				
Peak VO_2_, mL·kg^−1^·min^−1^	38.30 (34.00–43.30)	38.35 (34.00–44.00)	38.20 (33.95–42.75)	0.622
Peak HR, beats per minute	181 (176, 190)	181 (173, 187)	184 (176, 190)	< 0.001
Heart rate recovery after exercise test				
HRR∆ 2, beats per minute	44 (37–50)	45 (39–51)	43 (37–49)	< 0.001
HRR∆ 2, (% abnormity)	567 (45.30)	226 (40.10)	341 (49.50)	0.001
Sedentary time and physical activity				
Total sedentary time, hour·day^−1^	6.14 (4.67–7.86)	4.50 (3.45–5.21)	7.57 (6.57–8.71)	< 0.001
Moderate-to-vigorous physical activity				
Low MVPA (%)	291 (23.20)	91 (16.10)	200 (29.00)	< 0.001
Medium MVPA (%)	275 (21.90)	109 (19.30)	166 (24.10)	< 0.001
High MVPA (%)	687 (54.80)	364 (64.50)	323 (46.90)	< 0.001

Values are presented as the mean ± SD, median (25%–75%), or n (%)

*p* value based on Students t-test, Wilcoxon rank sum test or Chi square test

*Abbreviations: LST* low sedentary time, *HST* high sedentary time, *HRR* heart rate recovery, *MVPA* moderate-to-vigorous physical activity

### Associations of ST or MVPA with an abnormal HRR among healthy young and middle-aged adults

Table 2 shows the association between ST, MVPA and the incidence of an abnormal HRR through regression model analysis. Among healthy young and middle-aged adults, HST was significantly associated with higher odds of an abnormal HRR compared to LST; OR (95% CI) = 1.473 (1.172–1.852) in the fully adjusted model (Model 2). The medium MVPA was not significantly associated with a lower odds of an abnormal HRR compared with low MVPA (OR, 95% CI = 0.884, 0.631–1.238, Model 2). However, compared with low MVPA, high MVPA was significantly associated with a lower odds of an abnormal HRR (OR, 95% CI = 0.624, 0.470–0.827, Model 2) (Table 2).

**Table 2:** Logistic regression analysis of the association between ST, MVPA with incident abnormal heart rate recovery in healthy young and middle-aged adults

Variables	N	Crude Model	Model 1	Model 2
ST				
LST (< 6 h·day^−1^) (ref.)	564	1.00	1.00	1.00
HST (≥ 6 h·day^−1^)	689	1.465 (1.170,1.835) ^*^	1.473 (1.175,1.846) ^*^	1.473 (1.172,1.852) ^*^
MVPA				
Low MVPA (0–149 min·week^−1^) (ref.)	291	1.00	1.00	1.00
Medium MVPA (150–299 min·week^−1^)	275	0.870 (0.625,1.210)	0.854 (0.613,1.189)	0.884 (0.631,1.238)
High MVPA (≥ 300 min·week^−1^)	687	0.617 (0.468,0.813) ^*^	0.608 (0.461,0.802) ^*^	0.624 (0.470,0.827) ^*^

LST (< 6 h·day^−1^) as the reference group. Low MVPA (0–149 min·week^−1^) as the reference group

*p‐value less than 0.05

The crude model did not put in any covariate. **Model 1:** adjusted for age and sex (male/female). **Model 2:** adjusted for age, sex (male/female), current smoking (yes/no), current alcohol consumption (yes/no), late sleep (yes/no), family health history (hypertension, diabetes, heart disease), body mass index, fasting glucose, total cholesterol, low-density lipoprotein cholesterol, high-density lipoprotein cholesterol, triglycerides, systolic blood pressure, diastolic blood pressure

*Abbreviation*s: *ST* sedentary time, *LST* low sedentary time, *HST* high sedentary time, *MVPA* moderate-to-vigorous physical activity, *ref*. reference

### Joint analysis of ST and MVPA impact on an abnormal HRR among young and middle-aged adults

**Fig. 2:**
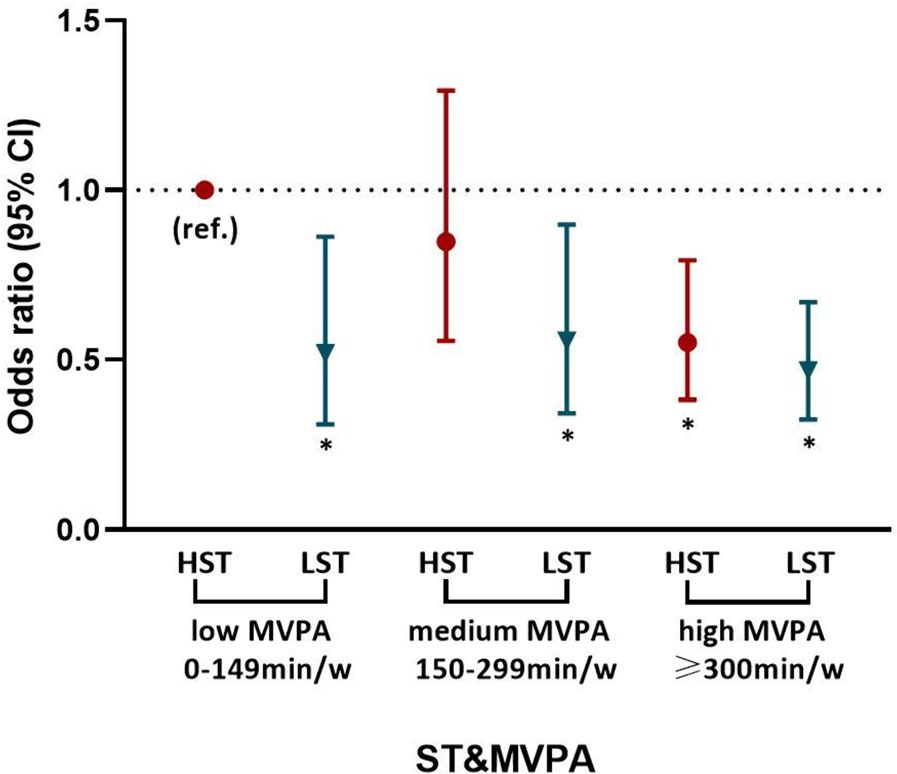
Adjusted joint associations of ST and MVPA with incident abnormal heart rate recovery in healthy young and middle-aged adults. Reference category for joint categories of sedentary time and moderate-to-vigorous physical activity is HST (≥ 6 h·day^−1^) and low MVPA (0–149 min·week^−1^). *: *p*‐value less than 0.05. Estimates based on Model 2. Adjusted for age, sex (male/female), current smoking (yes/no), current alcohol consumption (yes/no), late sleep (yes/no), family health history (hypertension, diabetes, heart disease), body mass index, fasting glucose, total cholesterol, low-density lipoprotein cholesterol, high-density lipoprotein cholesterol, triglycerides, systolic blood pressure, diastolic blood pressure. Abbreviations: LST = low sedentary time; HST = high sedentary time; MVPA = moderate-to-vigorous physical activity; ref. = reference

The association between the combined grouping of ST and MVPA with the odds of abnormal HRR in healthy young and middle-aged adults is shown in Table 3 and Fig. 2. Compared to the reference group (HST and low MVPA), those with HST and medium MVPA did not have lower odds of abnormal HRR (OR, 95% CI = 0.862, 0.566–1.312). Whereas, the HST and high MVPA group had lower odds of abnormal HRR compared to the reference group (HST and low MVPA) (OR, 95% CI = 0.553, 0.385–0.795). Furthermore, individuals with LST had a significantly lower odds of abnormal HRR compared to those with HST and low MVPA, regardless of whether the MVPA were low, medium, or high (OR, 95% CI = 0.515, 0.308–0.857 for LST and low MVPA; OR, 95% CI = 0.558, 0.345–0.902 for LST and medium MVPA; OR, 95% CI = 0.476, 0.326–0.668 for LST and high MVPA). Participants with LST and high MVPA had the lowest odds of abnormal HRR.

**Table 3:** Joint associations of ST and MVPA with incident abnormal heart rate recovery in healthy young and middle-aged adults

Variables	ST	
	HST (≥ 6 h·day^−1^)	LST (< 6 h·day^−1^)
MVPA	Crude Model	
low MVPA (0–149 min·week^−1^)	1.00 (ref.)	0.515 (0.308, 0.857) ^*^
medium MVPA (150–299 min·week^−1^)	0.849 (0.557, 1.295)	0.558 (0.345, 0.902) ^*^
high MVPA (≥ 300 min·week^−1^)	0.552 (0.384, 0.794) ^*^	0.476 (0.326, 0.668) ^*^
MVPA	Model 1	
low MVPA (0–149 min·week^−1^)	1.00 (ref.)	0.523 (0.316, 0.865) ^*^
medium MVPA (150–299 min·week^−1^)	0.820 (0.541, 1.242)	0.541 (0.337, 0.869) ^*^
high MVPA (≥ 300 min·week^−1^)	0.539 (0.377, 0.771) ^*^	0.459 (0.322, 0.653) ^*^
MVPA	Model 2	
low MVPA (0–149 min·week^−1^)	1.00 (ref.)	0.518 (0.311, 0.863) ^*^
medium MVPA (150–299 min·week^−1^)	0.848 (0.556, 1.293)	0.556 (0.344, 0.899) ^*^
high MVPA (≥ 300 min·week^−1^)	0.551 (0.383, 0.793) ^*^	0.468 (0.326, 0.670) ^*^

Reference category for joint categories of sedentary time and moderate-to-vigorous physical activity is HST (≥ 6 h·day^−1^) and low MVPA (0-149 min·week^−1^)

*p‐value less than 0.05

The crude model did not put in any covariate. **Model 1:** adjusted for age and sex (male/female). **Model 2:** adjusted for age, sex (male/female), current smoking (yes/no), current alcohol consumption (yes/no), late sleep (yes/no), family health history (hypertension, diabetes, heart disease), body mass index, fasting glucose, total cholesterol, low-density lipoprotein cholesterol, high-density lipoprotein cholesterol, triglycerides, systolic blood pressure, diastolic blood pressure. *Abbreviations: ST* sedentary time, *LST* low sedentary time, *HST* high sedentary time, *MVPA* moderate-to-vigorous physical activity, *ref* reference

### Dose–response relationship between MVPA and abnormal HRR stratified by ST level

**Fig. 3:**
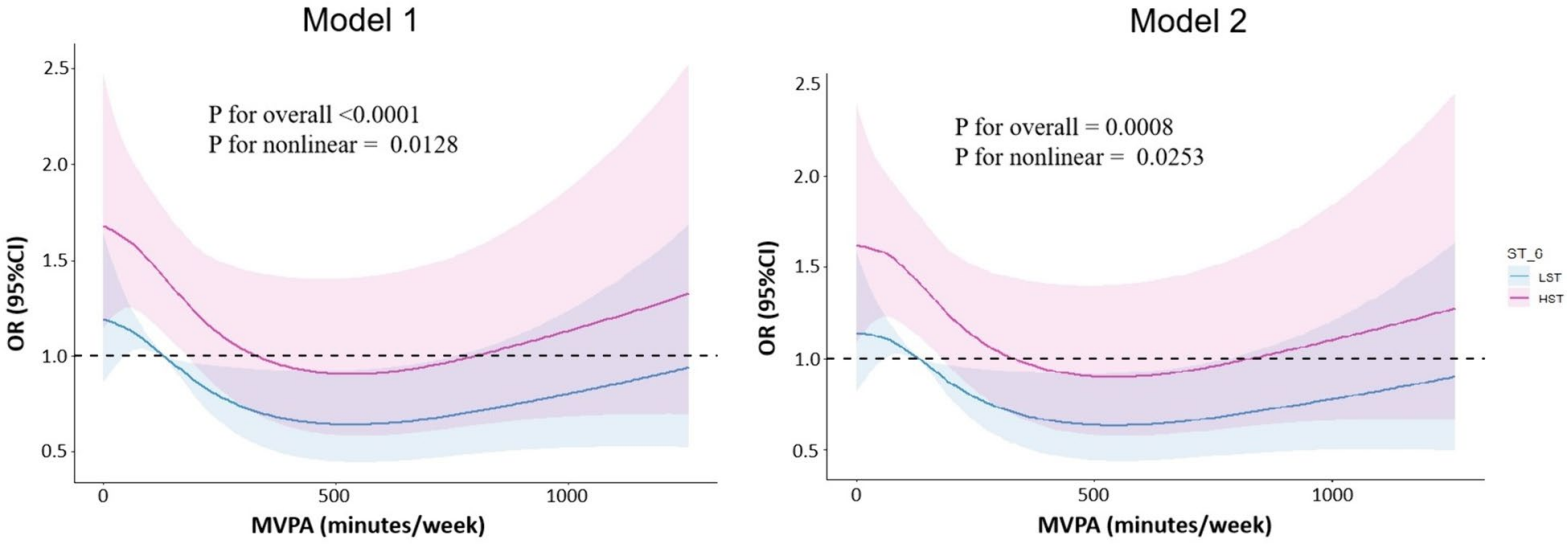
Dose–response relationship between MVPA with incident abnormal heart rate recovery in LST and HST groups. Model 1: Adjusted for age, sex (male/female). Model 2: Adjusted for age, sex (male/female), current smoking (yes/no), current alcohol consumption (yes/no), late sleep (yes/no), family health history (hypertension, diabetes, heart disease), body mass index, fasting glucose, total cholesterol, low density lipoprotein cholesterol, high density lipoprotein cholesterol, triglycerides, systolic blood pressure, diastolic blood pressure. Abbreviations: ST: sedentary time; LST = low sedentary time; HST = high sedentary time; MVPA = moderate-to-vigorous physical activity

The dose–response relationship between continuous MVPA (minutes·week^−1^) and the odds of abnormal HRR in LST and HST populations was studied by restricted cubic spline regression, as shown in Fig. 3. There was a non-linear association between MVPA time and the odds of abnormal HRR in HST and LST individuals (*P nonlinear* = 0.0253). After adjusting the potential confounding variables in model 2, for young and middle-aged adults with HST, when MVPA < 329 min·week^−1^, the odds of abnormal HRR is still significantly higher, but with the increase of MVPA time, the odds of abnormal HRR decreases; the odds of abnormal HRR was lower when MVPA was 329–829 min·week^−1^. Furthermore, for individuals with LST, the odds of abnormal HRR was significantly lower when MVPA exceeded 126 min·week^−1^.

## Discussion

A growing number of studies have shown that sedentary behavior was negatively associated with HRR in healthy adults [8, 9, 22]. Findings from our cross-sectional study confirmed that longer sedentary time has been associated with increased odds of abnormal HRR in healthy young and middle-aged adults. Nevertheless, it was also observed that higher amounts of MVPA, equivalent to participation in more than 300 min·week^−1^ of MVPA, may mitigate the negative impact of the longer sedentary time on HRR in this population.

MVPA was associated with lower cardiovascular events, morbidity, and mortality [30, 31]. Kiviniemi et al. [32] demonstrated there was a significant positive correlation between MVPA and HRR in healthy middle-aged adults after adjusting for confounders such as cardiopulmonary fitness and body fat percentage. These findings support the notion that PA benefits on cardiovascular health may be partly through modulation of autonomic activity. In addition, previous studies have shown that high-intensity intermittent aerobic exercise (without regular leisure exercise habits) can improve the heart rate recovery rate at 2 min after exercise more than continuous moderate-intensity aerobic exercise in sedentary adults [33]. Exercise may promote balance in the cardiac autonomic nervous system by increasing parasympathetic tone and decreasing sympathetic activity [34]. However, the nature of exercise interventions in the randomized controlled study [33] was different from the daily cumulative PA in the present study. Therefore, in this study, we focused on MVPA in daily life.

Prior studies have only separately investigated the correlation between sedentary behavior and HRR or PA and HRR. However, since the sedentary lifestyle is a phenomenon almost inevitable in modern society, especially among young and middle-aged adults, this combined analysis becomes extremely relevant.

Based on the World Health Organization's definition of sedentary behavior, a combined grouping of daily ST and MVPA was used in this study to further understand how much MVPA could attenuate or even counteract the adverse effects of sedentary activity on HRR. While more than 300 min·week^−1^ appeared to reduce the deleterious effects of sedentary activity on HRR in our study, this level of MVPA has been attested by large prospective cohort studies as necessary to mitigate sedentary-related increases in CVD mortality [20, 35]. Stamatakis et al. showed no significant correlation between being sedentary for more than 6 h per day and increased risk of cardiovascular mortality at a dose equivalent to meeting current recommendations for PA [35]. It is emphasized that engagement in more than 300 min·week^−1^ of MVPA is the latest World Health Organization recommended level of PA to promote greater health benefits in adults [18]. It is worth noting that for those who are sedentary for more than 6 h per day, the risk of abnormal HRR begins to increase after approximately more than 800 min of MVPA participation per week.This study provides further evidence of the detrimental effects of sedentary behavior on cardiovascular health and highlights the importance of MVPA in attenuating these risks, encouraging young and middle-aged adults to engage in more MVPA and, possibly, help to prevent the incidence of associated cardiovascular events. However, we should also consider the upper limit of the potential benefits of MVPA. Although increasing the duration of MVPA can be beneficial to some extent, there may be unfavourable physiological effects above a certain threshold. Therefore, it is essential to be cautious when designing exercise programs to ensure that individuals can reap the rewards of exercise without putting themselves at risk of over-exercising.

In this regard, it is known that an abnormal HRR is an independent risk factor for cardiovascular events [4, 5, 36]. Several large-scale population-based studies have investigated the joint association of ST and PA with other cardiovascular predictors, including maximal oxygen uptake, blood glucose, and triglyceride levels [37–40]. Nayor et al. [37] verified that adults with HST (≥ 821 min/day) and low VPA had the lowest levels of maximal oxygen uptake, and those with HST and high VPA had above-average maximal oxygen uptake. Another study conducted by Maranhao Neto et al. [38] showed found that adults with high TV time (≥ 4h/day) and inadequate PA were associated with higher blood sugar and triglyceride values. Still, independent of PA amounts, higher TV viewing time in adults was associated with higher values of adipose tissue. Furthermore, Alansare et al. [39], using an isotemporal substitution model, found that replacing sedentary time with 30 min of MVPA was a potential strategy for improving cardiac autonomic function as measured by heart rate variability in young and middle-aged women. The same research group [40] also showed a significant negative association between sedentary leisure time and heart rate variability by stratified analysis in a population of young and middle-aged women who did not meet the recommended leisure time for MVPA. It is relevant to point out that HRR and heart rate variability stand for different components of cardiac autonomic function [1, 2]. HRR reflects the changes in autonomic activity that occur immediately right after the exercise. The immediate decrease in heart rate after exercise mainly depends on parasympathetic activation [3]. Exercise and post-exercise recovery are high-risk periods for sudden death. Thus, HRR may have important predictive significance. Moreover, heart rate variability usually requires long time course monitoring, while HRR is less time-consuming than the former and, consequently, more accessible.

Corroborant with previous studies, the present study showed that HST remained significantly associated with increased odds of developing abnormal HRR in a healthy young and middle-aged population lacking MVPA. Previous studies demonstrated found that PA intensity is positively correlated with HRR [9]. Such results may be influenced by PA intensity or cardiovascular risk factor distribution. LST and low MVPA, LST and medium MVPA, LST and high MVPA had a significantly lower odds of abnormal HRR compared to those with HST and low MVPA. To some extent, the results indicate that the reduction of ST among those who participate in less than 300 min of MVPA per week. Notably, the present study highlights the negative effects of prolonged ST on abnormal HRR, but it has also been shown that regular breaks in prolonged ST can mitigate its potentially harmful effects [39]. This statement implies that we need to not only focus on reducing the total time spent sitting, but also encourage people to interrupt prolonged periods of sitting with PA. It is suggested that this could be further explored in subsequent studies. Taken together, the present study further complements related studies on the joint association of ST and PA with predictors of cardiovascular outcomes.

An online survey composed of 7753 adults found sedentary leisure behaviors increased while time spent in physical activity declined (*P* < 0.001) [41]. Another survey that enrolled 49,493 adults from 20 countries reported that the median time spent sitting was 360 min (quartiles 180–480 min) for younger adults [14]. The prevalence of individuals who adopt a sedentary style, reflected especially by the high sitting time spent, is increasing alarmingly, including in people who meet the recommended level of PA. Fighting this phenomenon can be considered one of the main challenges in public health today. An abnormal HRR is a meaningful predictor of the prevalence of cardiovascular system disease and all-cause mortality in healthy adults [4, 5, 36]. This study supports a referenceable exercise prescription for CVD and death prevention in healthy young and middle-aged adults aged 20–50 with sedentary behavior. It is consistent with current evidence showing that higher levels of PA could mitigate the adverse effects of sedentary behavior on abnormal HRR. This study only investigated whether MVPA could attenuate the adverse effects of sedentary activity on the occurrence of an abnormal HRR. Future studies could further compare the role of different intensities of PA in attenuating or even counteracting the adverse effects of excessive sedentary activity on HRR.

Nevertheless, it is essential to acknowledge the limitations of the present study. Firstly, as this is a cross-sectional study, it only suggests an association between ST and MVPA and abnormal HRR, not a causal relationship. Thus it is important to mention that prospective cohort study or randomized clinical study are necessary to confirm the influence of PA on HRR in individuals who spend long periods per day sitting. Secondly, the use of the IPAQ to assess ST and PA, while reliable, is not an objective assessing way, such as an accelerometer. Therefore, future studies should consider using objective measures to assess sedentary behavior and PA. Also, as the study's sample primarily consisted of Chinese participants, it is uncertain whether the findings generalize to other populations. It is hoped that more studies will be conducted in the future to evaluate other populations. Finally, this study only investigated whether the MVPA amount could attenuate the adverse effects of sedentary activity on the occurrence of abnormal HRR. Future studies could further compare the role of different intensities of PA in attenuating or even counteracting the adverse effects of excessive sedentary activity on HRR.

## Conclusion

Our joint analysis of ST, MVPA, and the odds of abnormal HRR showed that higher amounts of MVPA seemed to mitigate the negative impact of HST on HRR. Specifically, at least 300 min per week of MVPA may be needed to mitigate the hazardous effects of the HST on abnormal HRR in healthy young and middle-aged adults. Healthy young and middle-aged adults who cannot avoid sedentary time should be encouraged to meet the World Health Organization's recommendations on the level of PA to prevent future cardiovascular events. This study provides an opportunity for subsequent research comparing whether light, moderate, and high-intensity PA can mitigate the effects of sedentary activity on an abnormal HRR to increase understanding of the topic in this area.

### Supplementary Information

Supplementary Material 1.

Supplementary Material 2.

Supplementary Material 3.

## Data Availability

The datasets used and/or analyzed during the present study are available from the corresponding author on reasonable request.

## Abbreviations

HRR: Heart rate recovery
ST: Sedentary time
MVPA: Moderate-to-vigorous physical activity
IPAQ-LF: International Physical Activity Questionnaire-Long Form
LST: Low sedentary time
HST: High sedentary time
PA: Physical Activity
OR: Odds ratio
CI: Confidence interval
CVD: Cardiovascular disease
ECG: Electrocardiogram
VO_2_: Oxygen uptake
MET: Metabolic equivalents
CPET: Cardiopulmonary exercise test
HR: Heart rate
VO_2_peak: Peak oxygen uptake
HR_peak_: Peak heart rate
LDL cholesterol: Low-density lipoprotein cholesterol
HDL cholesterol: High-density lipoprotein cholesterol
BMI: Body mass index
