# Supplementary material for: Joint association of sedentary time and physical activity with abnormal heart rate recovery in young and middle-aged adults
Source: BMC Public Health. 2024 Jul 4;24:1787. doi: 10.1186/s12889-024-19298-9 (PMC11225313; doi:10.1186/s12889-024-19298-9)
Supplement: Supplementary file 1 — Supplementary Material 1. [file 12889_2024_19298_MOESM1_ESM.docx]

**Table S1** Logistic regression analysis of the association (OR and 95% CI) between ST, MVPA with incident abnormal heart rate recovery in healthy young and middle-aged adults.

|  | | incident abnormal heart rate recovery, OR (95% CI) | | |
| --- | --- | --- | --- | --- |
| Variables | N | Crude Model | Model 1 | Model 2 |
| **ST** | | | | |
| Men | 849 |  | | |
| LST (<6 hour·day^-1^) (ref.) | 374 | 1.00 | 1.00 | 1.00 |
| HST (≥6 hour·day^-1^) | 475 | 1.40 (1.07,1.84) **^*^** | 1.41 (1.07,1.85) **^*^** | 1.40 (1.06,1.84) **^*^** |
| Women | 404 |  | | |
| LST (<6 hour·day^-1^) (ref.) | 190 | 1.00 | 1.00 | 1.00 |
| HST (≥6 hour·day^-1^) | 214 | 1.51 (1.01,2.24) **^*^** | 1.63 (1.09,2.43) **^*^** | 1.62 (1.06,2.47) **^*^** |
| **MVPA** | | | | |
| Men | 849 |  | | |
| Low MVPA (0-149 minutes·week^-1^) (ref.) | 201 | 1.00 | 1.00 | 1.00 |
| Medium MVPA (150-299 minutes·week^-1^) | 193 | 0.870 (0.586, 1.292) | 0.861 (0.579, 1.281) | 0.868 (0.579, 1.301) |
| High MVPA (≥300 minutes·week^-1^) | 455 | 0.612 (0.438, 0.855) **^*^** | 0.607 (0.434, 0.848) **^*^** | 0.605 (0.429, 0.854) **^*^** |
| Women | 404 |  | | |
| Low MVPA (0-149 minutes·week^-1^) (ref.) | 90 | 1.00 | 1.00 | 1.00 |
| Medium MVPA (150-299 minutes·week^-1^) | 82 | 0.864 (0.474, 1.573) | 0.869 (0.475, 1.589) | 0.910 (0.484, 1.711) |
| High MVPA (≥300 minutes·week^-1^) | 232 | 0.634 (0.388, 1.035) | 0.620 (0.378, 1.016) | 0.593 (0.352, 0.999) **^*^** |

***** : *p*‐value less than 0.05.

Sedentary time <6 hour·day^-1^ as the reference (ref.) group.

Low MVPA (0-149 minutes·week-1) as the reference (ref.) group.

The crude model did not put in any covariate. Model 1: adjusted for age. Model 2: adjusted for age, current smoking (yes/no), current alcohol consumption (yes/no), late sleep (yes/no), family health history (hypertension, diabetes, heart disease), body mass index, fasting glucose, total cholesterol, low-density lipoprotein cholesterol, high-density lipoprotein cholesterol, triglycerides, systolic blood pressure, diastolic blood pressure.
