# Supplementary material for: Joint association of sedentary time and physical activity with abnormal heart rate recovery in young and middle-aged adults
Source: BMC Public Health. 2024 Jul 4;24:1787. doi: 10.1186/s12889-024-19298-9 (PMC11225313; doi:10.1186/s12889-024-19298-9)
Supplement: Supplementary file 2 — Supplementary Material 2. [file 12889_2024_19298_MOESM2_ESM.docx]

**Tables S2** Joint associations of sedentary time and moderate-to-vigorous physical activity (MVPA) with incident abnormal heart rate recovery in healthy young and middle-aged men adults.

| **Crude Model** | **sedentary time** | |
| --- | --- | --- |
|  | high sedentary time  (≥6 hour·day^-1^) | low sedentary time  (<6 hour·day^-1^) |
| **MVPA** |  | |
| low MVPA (0-149 minutes·week^-1^) | 1.00 (ref.) | 0.530 (0.321,0.876) **^*^** |
| medium MVPA (150-299 minutes·week^-1^) | 0.834(0.551,1.262) | 0.560 (0.350,0.898) **^*^** |
| high MVPA (≥300 minutes·week^-1^) | 0.551 (0.386,0.788) **^*^** | 0.467 (0.329,0.664) **^*^** |
| **Model 1** | **sedentary time** | |
|  | high sedentary time  (≥6 hour·day^-1^) | low sedentary time  (<6 hour·day^-1^) |
| **MVPA** |  | |
| low MVPA (0-149 minutes·week^-1^) | 1.00 (ref.) | 0.551 (0.301,1.009) |
| medium MVPA (150-299 minutes·week^-1^) | 0.799 (0.485,1.316) | 0.607 (0.347,1.062) |
| high MVPA (≥300 minutes·week^-1^) | 0.545 (0.355,0.839) **^*^** | 0.467 (0.304,0.716) **^*^** |
| **Model 2** | **sedentary time** | |
|  | high sedentary time  (≥6 hour·day^-1^) | low sedentary time  (<6 hour·day^-1^) |
| **MVPA** |  | |
| low MVPA (0-149 minutes·week^-1^) | 1.00 (ref.) | 0.550 (0.298,1.016) |
| medium MVPA (150-299 minutes·week^-1^) | 0.809 (0.488,1.342) | 0.610 (0.345,1.076) |
| high MVPA (≥300 minutes·week^-1^) | 0.539 (0.348,0.836) **^*^** | 0.470 (0.304,0.728) **^*^** |

***** : *p*‐value less than 0.05. Values are presented as OR (95% CI). Reference category for joint categories of sedentary time and physical activity is high sedentary time & low MVPA. The crude model did not put in any confounding factors. Model 1:adjusted for age; Model 2: Adjusted for age, current smoking (yes/no), current alcohol consumption (yes/no), late sleep (yes/no), family health history (hypertension, diabetes, heart disease), body mass index, glucose, total cholesterol, low density lipoprotein cholesterol, high density lipoprotein cholesterol, triglycerides, systolic blood pressure, diastolic blood pressure. OR, odds ratio; CI, confidence interval.
